# Injectable Versus Oral First-Line Disease-Modifying Therapies: Results from the Italian MS Register

**DOI:** 10.1007/s13311-020-01001-6

**Published:** 2021-02-02

**Authors:** Emanuele D’Amico, Aurora Zanghì, Marzia Romeo, Eleonora Cocco, Giorgia Teresa Maniscalco, Vincenzo Brescia Morra, Damiano Paolicelli, Giovanna De Luca, Simonetta Galgani, Maria Pia Amato, Giuseppe Salemi, Matilde Inglese, Paolo Agostino Confalonieri, Giacomo Lus, Carlo Avolio, Antonio Gallo, Marika Vianello, Marco Onofrj, Massimo Filippi, Maria Trojano, Francesco Patti

**Affiliations:** 1 Department “G.F. Ingrassia”, MS center, University of Catania, Policlinico G. Rodolico, V. Santa Sofia 78, 95123 Catania, Italy; 2 Neurorehabilitation Unit, IRCCS San Raffaele Scientific Institute, Milan, Italy; 3 Department “Medical Science and Public Health”, University of Cagliari, Cagliari, Italy; 4 Multiple Sclerosis Center and Neurological Clinic Stroke Unit , “A. Cardarelli” Hospital, Naples, Italy; 5 Multiple Sclerosis Center, University of Naples “Federico II”, Naples, Italy; 6 Department of Basic Medical Sciences, Neuroscience and Sense Organs, University of Bari “Aldo Moro”, Policlinico, Bari, Italy; 7 Neurology Unit, University G. D’Annunzio, Policlinico SS Annunziata, Chieti, Italy; 8 Multiple Sclerosis Center - Az. Osp. S. Camillo Forlanini, Rome, Italy; 9 Department NEUROFARBA University of Florence, Florence, Italy; 10 IRCCS Fondazione Don Carlo Gnocchi, Florence, Italy; 11 Department of Biomedicine, Neurosciences and Advanced Diagnostics, University of Palermo, Palermo, Italy; 12 Dipartimento Di Neuroscienze, Riabilitazione, Oftalmologia, Genetica E Scienze Materno - Infantili, Clinica Neurologica (DiNOGMI), Genoa, Italy; 13 Ospedale Policlinico San Martino, IRCCS, Genoa, Italy; 14 Fondazione IRCCS Istituto Neurologico Carlo Besta, Milan, Italy; 15 MS Center, II Division of Neurology, University della Campania “L. Vanvitelli”, Naples, Italy; 16 Centro Interdipartimentale Malattie Demielinizzanti, AOU Ospedali Riuniti Di Foggia, Foggia, Italy; 17 MS Center, I Division of Neurology, University della Campania “L. Vanvitelli”, Naples, Italy; 18 O.U. Neurology “Ca’ Foncello” Hospital – Treviso - MS Unit, Treviso, Italy; 19 Neurology Unit, Neurorehabilitation Unit, Neurophysiology Service, and Neuroimaging Research Unit, Division of Neuroscience, IRCCS San Raffaele Scientific Institute, Milan, Italy; 20 Vita-Salute San Raffaele University, Milan, Italy

**Keywords:** Multiple sclerosis, injectable DMTs, oral DMTs, real-world setting, EDSS score

## Abstract

The current study aims to compare injectable and oral first-line disease-modifying therapies (DMTs) for time to first relapse, time to confirmed disability progression (CDP), and time to discontinuation using a cohort of relapsing remitting multiple sclerosis (RRMS) patients, with data extracted from the Italian MS Register. This multicenter, observational, retrospectively acquired, and propensity-adjusted cohort study utilized RRMS-naïve patients from the Italian MS Register who started either injectable or oral first-line DMTs between January 1, 2010, and December 31, 2017, to evaluate the impact on disability outcomes in patients. Enrolled patients were divided into two groups, namely the injectable group (IG) and the oral group (OG). Of a cohort of 11,416 patients, 4602 were enrolled (3919 in the IG and 683 in the OG). The IG had a higher rate of women (67.3% vs 63.4%, *p* < 0.05) and a lower mean age (36.1 ± 10.9 vs 38.9 ± 11.8, *p* < 0.001). The event time to first relapse demonstrated a lower risk in the OG (HR = 0.58; CI 95% 0.48–0.72, *p* < 0.001). However, no differences were found between the two groups with respect to the risk of CDP (HR = 0.94; CI 95% 0.76–1.29, *p* = 0.941), while a lower risk of DMT was found in the OG (HR = 0.72; CI 95% 0.58–0.88, *p* = 0.002) for the event time to discontinuation. Real-world data from the Italian MS Register suggests that first-line oral DMTs are associated with a lower risk of experiencing a new relapse and of therapy discontinuation compared to injectable DMTs.

**Supplementary Information:**

The online version contains supplementary material available at 10.1007/s13311-020-01001-6.

## Introduction

Multiple sclerosis (MS) therapies have changed considerably over the last several decades, with the approval of oral disease-modifying therapies (DMTs) following the demonstration of efficacy and safety for the treatment of the relapsing forms of MS (RRMS) [1].

Prior to 2010, only DMTs administered by injection were available as an initial therapeutic option. Later, two oral drugs were approved in European countries as first-line DMTs: delayed release dimethyl fumarate (DMF), also known as gastro-resistant DMF, and teriflunomide (TRF) [2, 3]. Pivotal trials demonstrated the benefits of both DMF and TRF with clinical (i.e., number of clinical relapses and disability accrual) and magnetic resonance imaging (MRI) disease activity, with a generally good safety profile [4–11]. Real-world evidence (RWE) studies have shown that treatment with DMF and TRF controlled similar disease activity (assessed by no evidence of disease activity, NEDA-3, and time to the new first clinical relapse at a 12-month follow-up) and that the two DMTs showed comparable discontinuation rates at a 24-month follow-up [12, 13].

Established efficacy evidence for reducing relapsing activity and disability progression is available for all licensed first-line DMTs, but the need for real data of comparison between the established and the newer first-line DMTs in unselected patient populations is still needed to define treatment sequences and to gather real-world data on long-term outcomes [8–11, 14–19].

In the last several years, the Italian MS Register, the largest national clinical database with about 140 Italian MS centers, offered the opportunity to study real-world clinical outcomes in large cohorts of patients to represent daily clinical practice [18, 20].

The aim of the current study was to evaluate long-term outcomes of first-line DMTs in terms of time to first relapse, time to confirmed disability progression (CDP), and, additionally, time to discontinuation in RRMS-naïve patients by focusing on the direct comparison between injectable and oral first-line DMTs, namely interferons and glatiramer acetate compared to dimethyl fumarate and teriflunomide [18].

## Methods

### Population

A multicenter, observational, retrospectively acquired cohort study was utilized for the current study. Anonymized clinical data of patients with RRMS were extracted from the Italian MS Register from their first treatment prescription with injectable and oral DMTs (between January 1, 2010 and December 31, 2017) to their last follow-up with the same treatment [20].

Key eligibility criteria included (1) a diagnosis of RRMS according to the 2010 McDonald criteria [21]; (2) aged between 18 to 55 years at the time of first DMT prescription; (3) start of injectable or oral first-line DMTs between January 1, 2010, and December 31, 2017; (4) continuous exposure to the investigated DMTs for ≥ 6 months; and (5) patients with at least three visits (including baseline) with an Expanded Disability Status Scale evaluation (EDSS).

RRMS-naïve patients who matched the required criteria were divided into two groups for the analyses, the injectable group (IG) and oral group (OG). The IG included RRMS patients who were treated with either Copaxone (40 mg per ml/three times per week subcutaneously and at least 48 h apart) or IFNs (interferon β-1a and interferon β-1b, 30 μg/0.5 mL, once weekly, intramuscularly or interferonβ-1a, either 22 mcg or 44 mcg, three times per week subcutaneously) [22–25]. The OG included RRMS patients who were treated with either DMF (120 mg twice per day for the first 7 days, then 240 mg twice per day) or TRF (14 mg once per day) [2, 3].

### Study Endpoints

The primary study outcome was the evaluation of time to first relapse and time to CDP. The time interval from baseline to the first event (for patients with an event) or to the last evaluation at follow-up (for patients without an event) was examined. Additionally, the time to discontinuation of the first prescribed DMT was evaluated.

### Procedures and Outcomes

Patients were included in the study at the initiation of treatment (baseline) and were monitored over their full time on the medication with data collection performed at baseline and approximately every 6 months during the time of exposure. Patients were censored at treatment discontinuation or at their last recorded clinical visit.

A relapse was defined as new symptoms or an exacerbation of existing symptoms persisting for ≥ 24 h in the absence of concurrent illness/fever and occurring ≥ 30 days after a previous relapse. CDP events were defined as ≥ 6-month confirmed increases of either ≥ 0.5 points for patients with a baseline EDSS score > 5.5, ≥ 1.0 point for those with a baseline EDSS score of 1 and 5.5, and ≥ 1.5 points for those with a baseline EDSS score of 0. A minimum of three visits, including the baseline visit, with an EDSS score evaluation, was required. EDSS scores recorded within 30 days after the onset of a relapse were excluded.

Discontinuation of investigated drugs was defined as a gap of treatment for 60 or more days. Time to discontinuation (in months) was measured as the time between the index date and the end of the supply of the prescriptions dispensed.

### Statistical Analyses

Data are presented as counts (proportions) for categorical variables and mean (standard deviation, SD) or median (interquartile range, IQR) for continuous variables. Unpaired *t* tests and Mann Whitney tests were used to compare continuous variables according to their distribution. Chi-square tests were used to compare categorical variables. Univariate non-parametric Kaplan Meier (K-M) curves and log-rank tests were used to evaluate the events under investigation in the entire sample.

A Schoenfeld’s global test was used to verify the proportional hazards assumption for the time-on-treatments. Once the proportionality assumption was verified, a Cox proportional model was built for each investigated outcome in the entire sample. A Cox proportional hazard univariate regression model was used to estimate the hazard ratio (HR) and its 95% confidence interval (CI).

To take into account the imbalance of the two groups, a propensity score (PS) was calculated.

Additionally, a logistic regression was conducted to evaluate all patients using treatment (injectable vs oral) as the independent variable and baseline levels of sex, age, type of MS onset (monofocal or multifocal), EDSS score, number of relapses in the year prior to onset, and disease duration as covariates. Inverse probability of treatment weight (IPTW) and the stabilized inverse probability of treatment weight (SIPTW) were also calculated.

Standardized differences calculated in weighted (using the stabilized weights) and unweighted samples were used to assess the balance of baseline covariates between treated and control.

Multivariable Cox proportional hazard regression models weighted for IPTW were performed to evaluate the relationship between outcomes and treatment groups. HRs and 95% CIs were calculated to evaluate the relationship between outcomes and the treatment group.

For the analysis of relapse outcomes, a negative binomial model and weighted negative binomial model were conducted, using the annual relapse rate as the dependent variable and group as the independent variable.

To better examine the differences between the two treatment strategies in mild-to-moderate patients, subgroup univariate analyses were conducted, stratifying patients on baseline EDSS scores (≤ 2 and > 2) and for number of relapses in the pre-baseline year (1 or more than 1), and a Cox proportional hazard univariate regression model was applied to each subgroup. HRs and their 95% CIs were reported. A sensitivity analysis was conducted on patients with at least 30 months of follow-up.

Furthermore, as a method of correcting the variables that were not measured, the E-value proposed by VanderWeele et al. was calculated [26]. The E-value was defined as the minimum strength of association on the hazard ratio scale that an unmeasured confounding variable would need to have with both the group and the outcome to fully explain the specific group-outcome association, conditional on the measured covariates.

Missing data were handled through multiple imputation. The analysis used normalized weights to approximate the inferences in the data with data missing not at random (MNAR) [27]. The associations between missingness of the baseline data and other demographical and clinical characteristics were calculated with a multivariate logistic regression analysis, as previously published [28, 29].

All results were considered significant at 0.05. Stata 16.1 was used for all analyses.

### Protocol Approvals Standard, Registrations, and Patient Consents

Use of the Italian MS Register was approved by the Ethics Committee of the University of Bari (Italy) as the coordinator center (Reference numbers 0055587 and 0052885) and by the local Ethics Committees of all participant centers. The study protocol for the current analysis was also discussed and approved by the Scientific Committee of the Italian MS Register. Each subject enrolled with a diagnosis of MS was required to sign written informed consent to enter the Register. In some centers in which data had been collected before the Register was set up, depending on local laws and regulations, historical data collected retrospectively were also included without informed consent when the patient was not traceable due to death, transfer, or for other reasons. The current report does not contain any individual or identifying information.

### Data Availability

Anonymized data will be shared by request from a qualified investigator for the sole purpose of replicating procedures and results presented in the report, provided that the data transfer is in agreement with EU legislation on the general data protection regulation.

## Results

### Participants

**Fig. 1:**
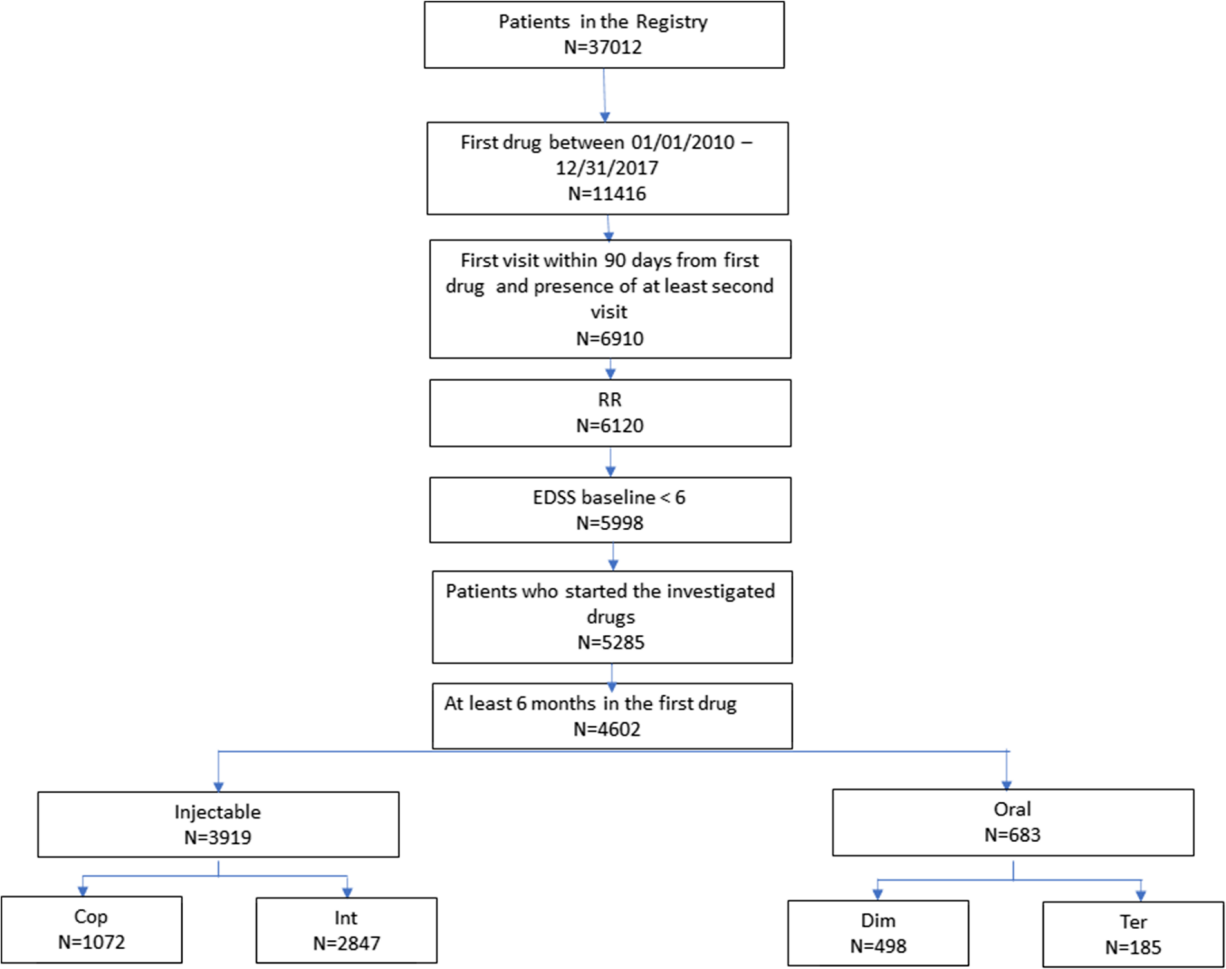
Patients’ selection flow chart. DMT, disease-modifying therapies; EDSS, Expanded Disability Status Scale; IG, injectable group; OG, oral group; RRMS, relapsing remitting multiple sclerosis

Out of a cohort of 37,012 patients selected from the Italian MS Register, 11,416 started their first DMT during the index window. Out of those subjects, 4602 (3919 in the IG and 683 in the OG) were considered eligible for the analyses and were subsequently enrolled (Fig. 1).

Baseline characteristics by group are shown in Table 1. Patients in the IG had a higher rate of women (67.3% vs 63.4%, *p* < 0.05) and a lower mean age (36.1 ± 10.9 vs 38.9 ± 11.8, *p* < 0.001, see Table 1).

**Table 1:** Baseline characteristics of the two groups

	IG		OG			Unweighted standardized mean differences	Weighted standardized mean differences
*N*	3919		683		*p*		
Female (*n*, %)	2639	67.3	433	63.4	0.044	0.08	− 0.01
Age (mean, sd) (year)	36.1	10.9	38.9	11.	< 0.001	− 0.25	0.01
Monofocal onset (*n*, %)	3750	95.7	649	95	0.434	0.03	− 0.0003
EDSS at baseline (median, q1–q3)	1.5	1–2	1.5	1–2.5	0.245	− 0.07	0.01
Relapses in the year before treatment start (*n*, %)	2763	70.5	462	67.6	0.132	0.06	0.02
ARR (mean, sd)*	1.4	0.6	1.3	0.7	0.388	0.04	0.01
DD (median, q1–q3)	66	23–249	70	21–277	0.0001	− 0.12	0.01

*Only in patients with relapses in the last year

*ARR*, annualized relapse rate; *EDSS*, Expanded Disability Status Scale; *IG*, injectable group; *OG*, oral group; *DD*, disease duration

Median disease duration was longer in the IG (66 months with a range of 23–249 compared to 70 months with a range of 21–277, *p* < 0.0001).

The median follow-up of the total cohort was 36 months (IQR = 22–36 months), while the follow-up in the IG was a median of 36 months (IQR = 28–36 months) and the OG median was 19 months (IQR = 12–27 months), *p* < 0.0001.

### Findings on the Pre-matched Samples

**Fig. 2:**
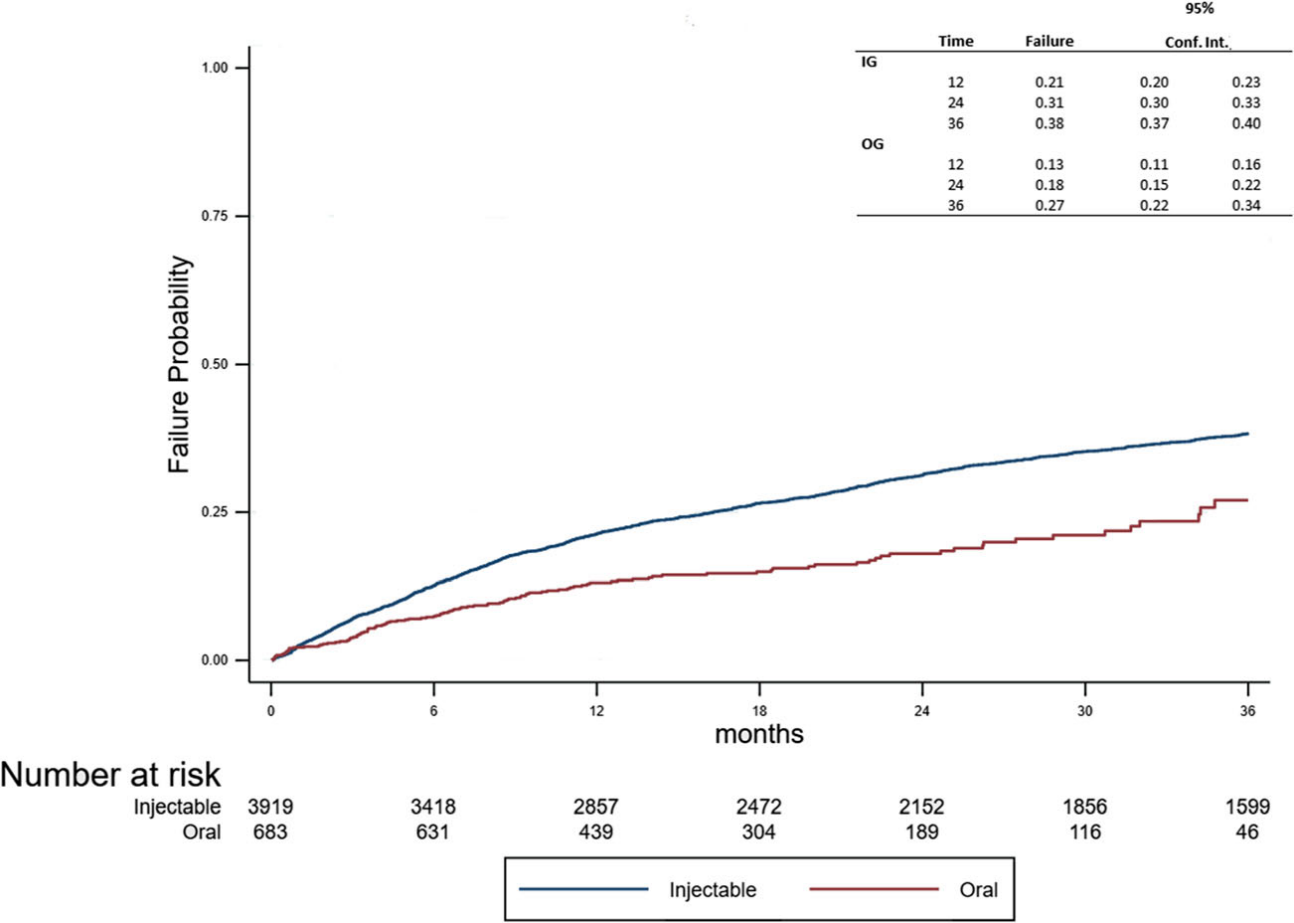
Time to first relapse between the two groups

During the follow-up, 1477 patients relapsed (*n* = 1363 (35%) in the IG, *n* = 114 (17%) in the OG). A log-rank test demonstrated that the risk to experience the first relapse was lower in the OG (*p* < 0.001, Fig. 2), which was also confirmed by the Cox model (HR = 0.57; CI 95%: 0.47–0.69).

A negative binomial model and weighted negative binomial model were applied using the annual relapse rate as the dependent variable and group (IG vs. OG) as the independent variable. The incidence rate ratio (OG vs. IG) was 0.63 (95% CI: 0.51–0.78, *p* < 0.001). After an inverse probability weighting, the incidence rate ratio was 0.65 (95% CI: 0.52–0.82, *p* < 0.001). Taking this into consideration with the total count of relapses, the OG demonstrated a lower risk than the IG.

**Fig. 3:**
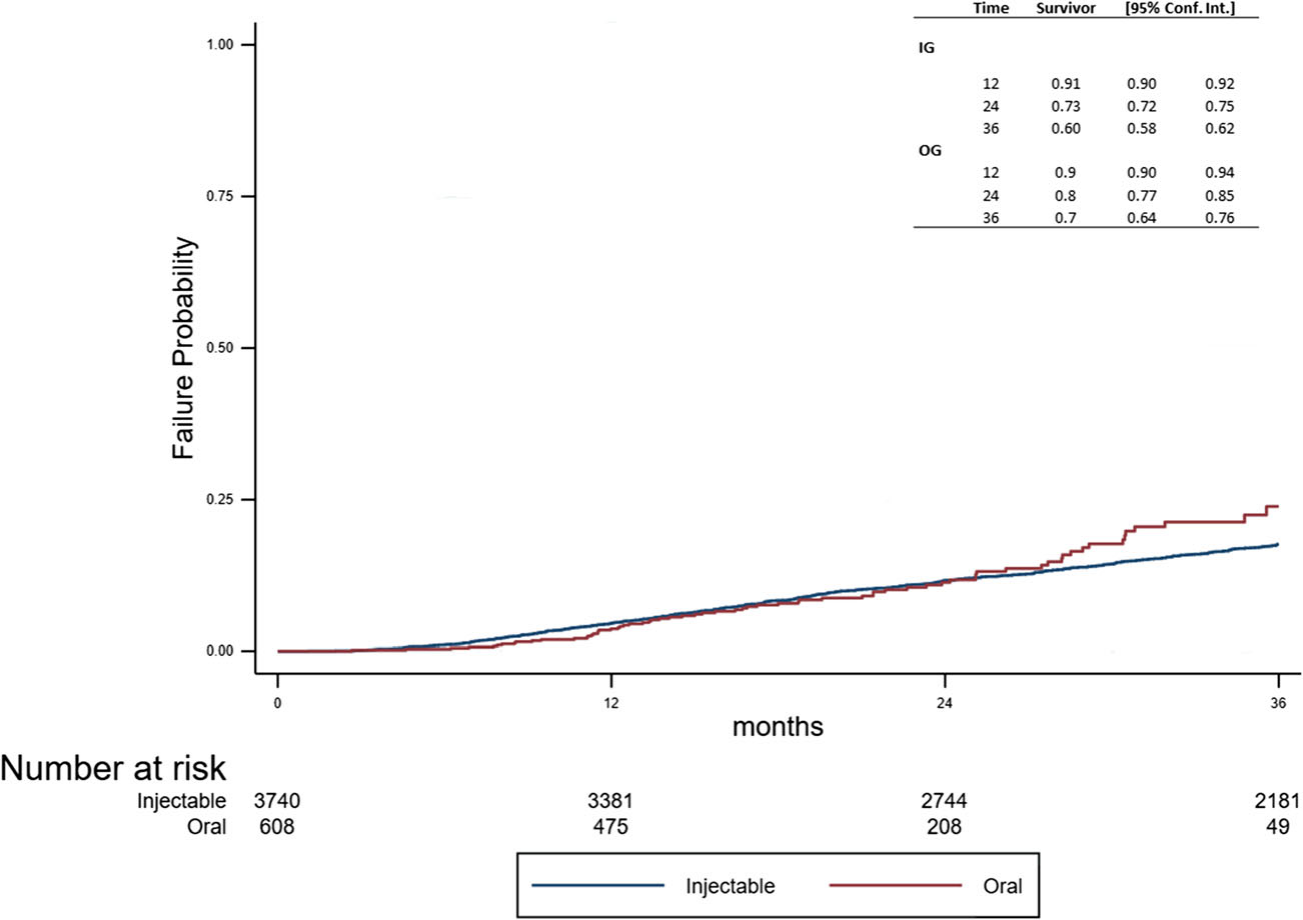
Time to CDP between the two groups. CDP, confirmed disability progression

The event CDP was observed in 641 patients (*n* = 574 (15%) in the IG, *n* = 67 (10%) in the OG). The risk to reach CDP did not differ between the two groups using a log-rank test (*p* = 0.370, Fig. 3). This was confirmed by the Cox model (HR = 1.12; 95% CI: 0.87–1.45, *p* = 0.370).

**Fig. 4:**
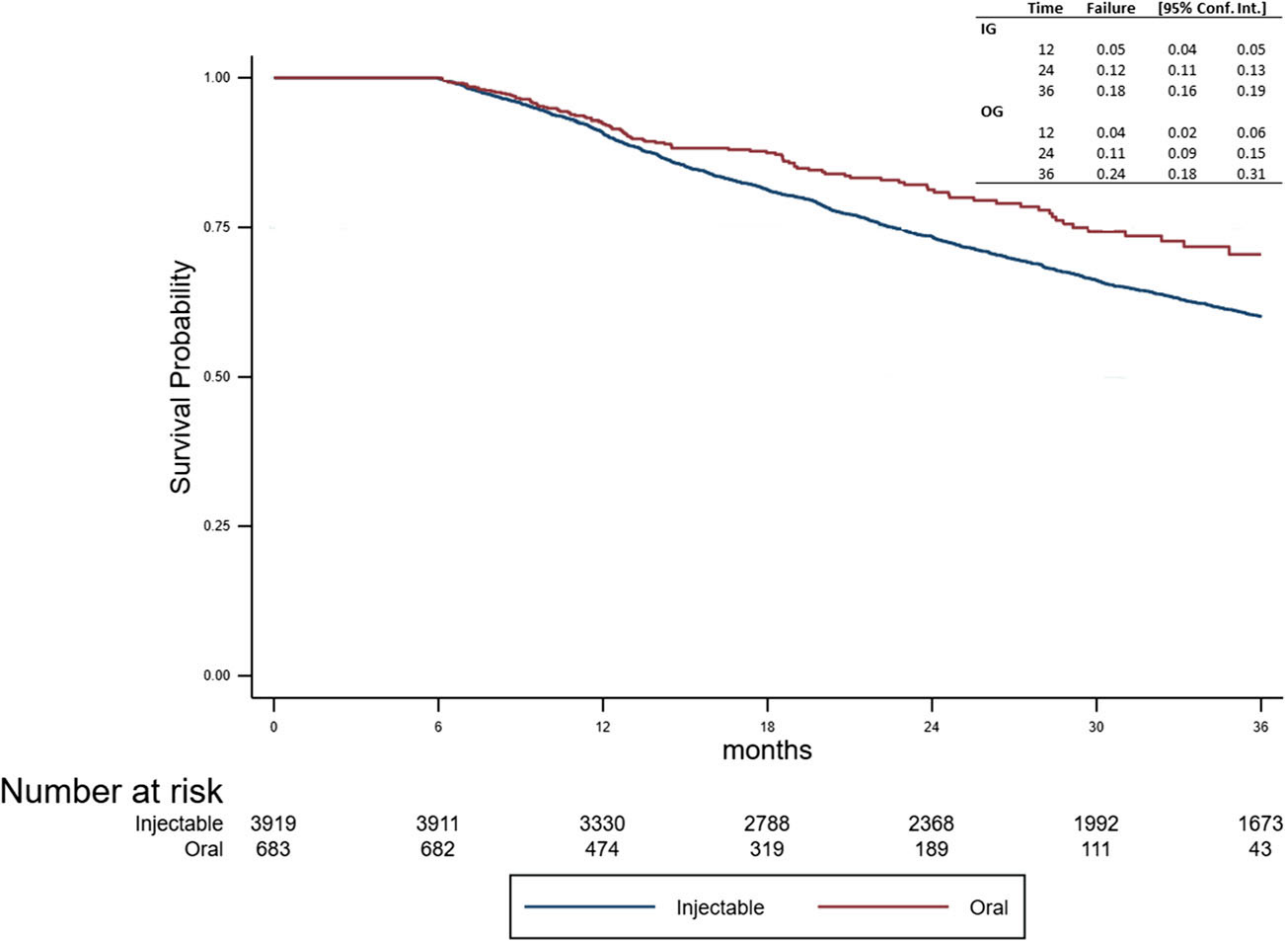
Time to first DMT discontinuation between the two groups

Finally, DMT discontinuation was observed in 1456 patients (*n* = 1352 (34.5%) in the IG, *n* = 104 (15%) in the OG). The risk of DMT discontinuation was lower in the OG as per the log-rank test (*p* < 0.001), which was confirmed by the Cox model (HR = 0.71; 95% CI: 0.58–0.86, Fig. 4).

### Findings After IPW PS Adjustment

**Fig. 5:**
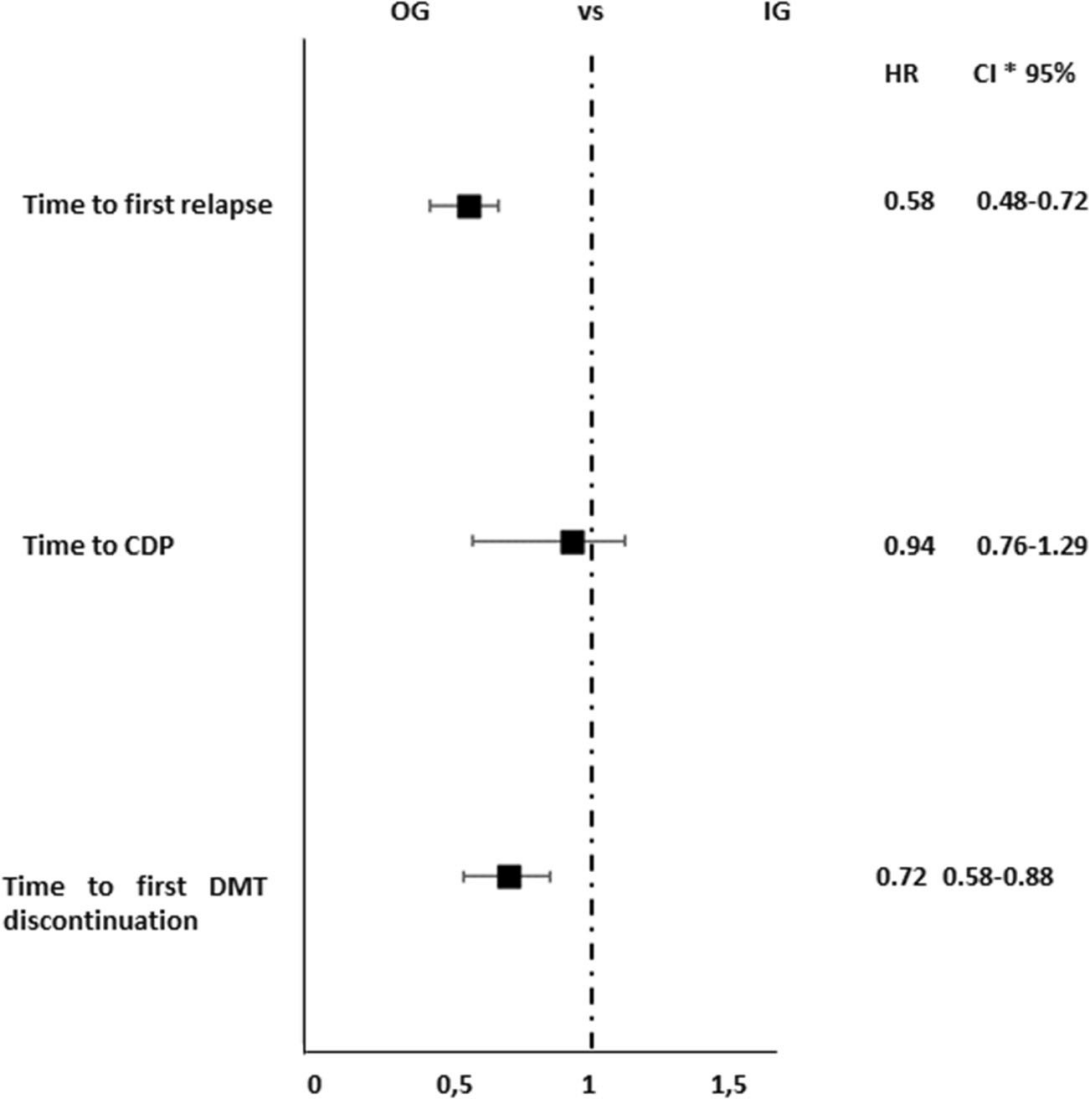
Analysis of treatment effects in time to first relapse, time to CDP, and time to DMT discontinuation. (Asterisk) The treatment effects were explored by a propensity score adjustment in quintiles for age, duration of disease from onset, Expanded Disability Status Scale at baseline, relapses in the previous year, sex, and clinical onset. CI, confidence interval; HR, hazard ratio; CDP, confirmed disability progression; DMT, disease-modifying therapies; IG, injectable group; OG, oral group

Results from the Cox models following the IPW PS adjustment were consistent with the previous models (Fig. 5). The event time to first relapse demonstrated a lower risk in the OG (HR = 0.58; 95% CI: 0.48–0.72, *p* < 0.001). No differences were found for the risk of CDP between the two groups (HR = 0.94; 95% CI: 0.76–1.29, *p* = 0.941). However, a lower risk of DMT discontinuation was found in the OG (HR = 0.72; 95% CI: 0.58–0.88, *p* = 0.002, Fig. 5).

### Subgroup Analyses

Subgroup analyses were also performed for each investigated outcome to examine any difference in the two groups in mild-to-moderate patients. IG and OG subjects were stratified based on their baseline EDSS score (≤ 2 or > 2) and the number of relapses during the year prior to their baseline visit (1 or > 1, Table 2).

**Table 2:** Subgroup univariate analysis of treatment effects in terms of risk of first relapse, CDP, and DMT discontinuation

IG vs OG					
	IG	OG	HR	CI 95%	*p*
First relapse					
EDSS ≤ 2	2971	493	0.63	0.50–0.79	< 0.001
EDSS>2	948	190	0.44	0.31–0.63	< 0.001
Relapses in the year pre-baseline (*n* = 1)	3094	553	0.53	0.42–0.67	< 0.001
Relapses in the year pre-baseline (*n* > 1)	825	130	0.75	0.54–1.06	0.103
CDP					
EDSS ≤ 2	2836	438	1.06	0.77–1.46	0.707
EDSS > 2	904	170	1.22	0.79–1.88	0.364
Relapses in the year pre-baseline (*n* = 1)	2941	489	1.23	0.93–1.63	0.141
Relapses in the year pre-baseline (*n* ≥ 1)	799	119	0.76	0.40–1.46	0.417
DMT discontinuation					
EDSS ≤ 2	2971	493	0.64	0.50–0.82	< 0.001
EDSS > 2	948	190	0.85	0.61–1.19	0.342
Relapses in the year pre-baseline (*n* = 1)	3094	553	0.75	0.60–0.94	0.014
Relapses in the year pre-baseline (*n* ≥ 1)	825	130	0.58	0.37–0.92	0.020

*CDP*, confirmed disability progression; *DMT*, disease-modifying therapies; *EDSS*, Expanded Disability Status Scale; *IG*, injectable group; *N*, number; *OG*, oral group

The OG had lower risk of event time to first relapse than the IG, for both EDSS subgroups and the subgroup with one relapse in the year prior to baseline (Table 2). Furthermore, the risk of discontinuation was lower in the OG for subjects with an EDSS ≤ 2 and for both relapse subgroups (Table 2). There were no statistically significant differences found between the two groups for CDP.

### Sensitivity Analysis

A sensitivity analysis was conducted on 3007 (139 OG and 2868 GI) subjects with at least 30 months (out of 36) of follow-up. The interquartile range around the median at follow-up visits became much smaller, with median follow-up of 36 months (IQR = 36–36 months). In the IG, the median was also 36 months (IQR = 36–36 months), while in the OG it was 35 months (IQR = 33–36 months).

Before the PS adjustment, hazard ratios were obtained for first relapse (HR = 0.48; 95% CI: 0.34–0.70, *p* < 0.001), for time to CDP (HR = 1.11; 95% CI: 0.74–1.68, *p* = 0.613), and for time to first DMT discontinuation (HR = 0.61; 95% CI: 0.43–0.87, *p* = 0.006). After IPW PS adjustment, hazard ratios were also obtained for time to first relapse (HR = 0.50; 95% CI: 0.35–0.73, *p* < 0.001), for time to CDP (HR = 1.12; 95% CI: 0.74–1.69, *p* = 0.604), and for time to first DMT discontinuation (HR = 0.62; 95% CI: 0.44–0.89, *p* = 0.009).

### E-Values for Unmeasured Variables

The observed HRs could be explained away by an unmeasured confounding variable that was associated with in the observed group and the outcome by a E-value of 2.27 for time to relapse, 1.26 for CDP, and 1.82 for DMT discontinuation, but a weak confounding variable could not do this.

## Discussion

In this multicenter, observational, retrospectively acquired cohort study, starting oral first-line DMTs (DMF and TRF) was associated with a lower risk of first relapse occurrence and treatment discontinuation rate during the follow-up, in comparison to first-line injectable DMTs, but no significant difference was found in reaching CDP.

In clinical practice, oral DMTs are gradually replacing the injectable DMTs after they were licensed for RRMS treatment because of their improved tolerability by the patients [30]. However, injectable DMTs have been thoroughly investigated in terms of efficacy and are still largely prescribed for their well-characterized safety profile. Moreover, they are still widely considered for patients who intend to become pregnant, having been approved for use during childbearing and breastfeeding (which was recently extended for IFNs) [31, 32].

First-line injectable and oral DMTs have not been compared in non-inferiority trials, nor have they been compared in registry based cohorts studies as first choice DMTs [33, 34].

Considering that big data registries offer the opportunity to study real-world clinical outcomes in large cohorts of patients, the strengths of the current work include the generalizability, the representation of daily clinical MS practice, and the large cohort of patients collected in the Italian MS Register, which is the largest national clinical database with about 140 Italian MS centers connected [20, 35].

The current data suggest that first-line oral DMTs should be a suitable first choice in RRMS patients when, according to a prognostic profile, a first-line DMT is required.

Patient and disease heterogeneity at the initial presentation and during the disease course makes the increasing treatment choices for RRMS valuable, thereby allowing for the personalization of treatment. First, long-term results of the drug safety and efficacy of a compound may inform decision-making. Here, real-world data and well-structured registries are of importance, as is the statistical method employed. PS adjustment allowed for the mitigation of the effect of heterogeneity of the data. However, all PS methods cannot eliminate bias due to unknown or unmeasured confounding variables. Since an observational retrospective study has biases related to data collection, in particular, in the two groups demonstrating a difference in the follow-up period, the survival analysis allows this to remain under control. Moreover, the sensitivity analysis conducted was limited to patients with at least 30 months of follow-up. The possible effect of unmeasured confounding variables was calculated using an E-value [36–39].

The primary limitations of our study pertain to the observational nature of the data. In addition, MRI activity was not evaluated, nor was there a correction for MRI parameters in the PS model. This may be a limitation, as the current criteria for defining the efficacy of a treatment of MS use composite scores, as no evidence of disease activity (NEDA3) must take into account the presence of new or active (enhancing) lesions on MRI scans [40]. This is, indeed, missing in many real-world studies. The level of evidence of a descriptive study without MRI data is limited and cannot replace a non-inferiority trial.

The current study was not designed to compare the safety of the two approaches because the safety data were not sufficiently complete to enable such an analysis. Further research is needed to more accurately identify patients who are most likely to benefit from these therapies.

### Supplementary Information

ESM 1 (PDF 1899 kb)

## Appendix 1

**Table 3:** Authors

Name	Location	Role	Contribution
Emanuele D’Amico	Department G.F. Ingrassia, University of Catania, Italy	Author	Study concept and design, acquisition of data, supervision
Aurora Zanghì	Department G.F. Ingrassia, University of Catania, Italy	Author	Study concept and design, acquisition of data, supervision
Marzia Romeo	Neurology Dept. and INSPE-Institute of Experimental Neurology, Vita-Salute San Raffaele Univ., Milan	Author	Analysis and interpretation of data
Eleonora Cocco	Centro Regionale Per La Diagnosi E La Cura Della Sclerosi Multipla ASL8 P.O. Binaghi, Cagliari, Italy	Author	Study concept and design, acquisition of data, supervision
Giorgia Teresa Maniscalco	Regional Center of Diagnosis and Therapy of Multiple Sclerosis, Naples,Italy	Author	Study concept and design, acquisition of data, supervision
Vincenzo Bresciamorra	AOU Policlinico Federico II, Naples, Italy	Author	Study concept and design, acquisition of data, supervision
Damiano Paolicelli	Dept. of Basic Medical Sciences, Neuroscience and Sense Organs, Univ. of Bari “Aldo Moro”, Policlinico, Bari, Italy	Author	Study concept and design, acquisition of data, supervision
Giovanna De Luca	Neurology Unit, Univ. G. D’Annunzio, Policlinico SS Annunziata Chieti, Italy	Author	Study concept and design, acquisition of data, supervision
Simonetta Galgani	Centro Sclerosi Multipla - Az. Osp. S. Camillo Forlanini, Roma, Italy	Author	Study concept and design, acquisition of data, supervision
Maria Pia Amato	Dept. NEUROFARBA Univ. of Florence, MS Center AOU Careggi, Florence, Italy	Author	Study concept and design, acquisition of data, supervision
Giuseppe Salemi	MS centre, Dept of Emergency, Urgency and Neuroscience, Univ. of Palermo, Italy	Author	Study concept and design, acquisition of data, supervision
Matilde Inglese	Dipartimento Di Neuroscienze, Riabilitazione, Oftalmologia, Genetica E Scienze Materno - Infantili, Clinica Neurologica - Ospedale Policlinico San Martino (DiNOGMI), Italy	Author	Study concept and design, acquisition of data, supervision
Paolo Agostino Confalonieri	Fondazione IRCCS Istituto Neurologico Carlo Besta, Milan, Italy	Author	Study concept and design, acquisition of data, supervision
Giacomo Lus	MS Center, II Division of Neurology, Univ. della Campania “L. Vanvitelli”, Naples, Italy	Author	Study concept and design, acquisition of data, supervision
Carlo Avolio	Centro Interdipartimentale Malattie Demielinizzanti - AOU Ospedali Riuniti Di Foggia	co-investigator	Acquisition of data
Antonio Gallo	MS Center, I II Division of Neurology, Univ. della Campania “L. Vanvitelli”, Naples, Italy	Author	Study concept and design, acquisition of data, supervision
Marika Vianello	Neurology “Ca′ Foncello” Hospital – Treviso - MS Unit, Italy	Author	Study concept and design, acquisition of data, supervision
Marco Onofrj	Neurology Unit, Univ. G. D’Annunzio, Policlinico SS Annunziata Chieti, Italy	Author	Study concept and design, acquisition of data, supervision
Massimo Filippi	Neuroimaging Research Unit, Institute of Experimental Neurology, Division of Neuroscience, San Raffaele Scientific Institute, Vita-Salute San Raffaele University, Milan, Italy.	Author	Study concept and design, acquisition of data, supervision
Maria Trojano	Dept. of Basic Medical Sciences, Neuroscience and Sense Organs, Univ. of Bari “Aldo Moro”, Policlinico, Bari, Italy	Author	Study concept and design, acquisition of data, critical revision of manuscript for intellectual content
Francesco Patti	Department G.F. Ingrassia, University of Catania, Italy	Author	Study concept and design, acquisition of data, critical revision of manuscript for intellectual content

## Appendix 2

**Table 4:** Co-investigators for Italian MS register

Name	Location	Role	Contribution
Francesco Passantino	Divisione Di Neurologia - Azienda Ospedaliera SS. Antonio E Biagio E Cesare Arrigo	Co-investigator	Acquisition of data
Maura Chiara Danni	Centro SM - Clinica Neurologica - Ospedali Riuniti di Ancona	Co-investigator	Acquisition of data
Rocco Totaro	Centro Malattie Demielinizzanti - Clinica Neurologica, Ospedale San Salvatore - L’Aquila	Co-investigator	Acquisition of data
Maria Gabriella Coniglio	Centro Sclerosi Multipla P.O. Madonna delle Grazie	Co-investigator	Acquisition of data
Diomira Acquistapace	Centro Sclerosi Multipla - Azienda Ospedaliera Regionale S. Carlo	Co-investigator	Acquisition of data
Roberto Bruno Bossio	U.O. di Neurologia - Centro SM - ASP di Cosenza	Co-investigator	Acquisition of data
Paola Valentino	Centro Sclerosi Multipla - Policlinico Universitario - Campus Germaneto	Co-investigator	Acquisition of data
Carlo Pozzilli	S.Andrea MS Center, Sapienza Univ. Rome, Italy	Author	Study concept and design, acquisition of data, supervision
Sauro Severi	Ambulatorio SM - Sezione Neurologia - Ospedale Valdarno, Montevarchi (AR)	Co-investigator	Acquisition of data
Benedetta Calchetti	Centro Aziendale SM - U.O. Neurologia - Ospedale S. Donato, Arezzo	Co-investigator	Acquisition of data
Daniele Spitaleri	Centro Sclerosi Multipla - U.O.C. Neurologia, AORN San G. Moscati di Avellino	Co-investigator	Acquisition of data
Maurizia Gatto	Centro Malattie Demielinizzanti - Ospedale Generale Regionale F. Miulli	Co-investigator	Acquisition of data
Pietro Iaffaldano	Centro SM Dipartimento di Scienze Mediche di Base, Neuroscienze ed. Organi di Senso Universita’ di Bari	Co-investigator	Acquisition of data
Bonaventura Ardito	Centro Sclerosi Mutipla UOC Di Neurologia - Ospedale Della Murgia Fabio Perinei	Co-investigator	Acquisition of data
Valeria Barcella	Centro Provinciale Sclerosi Multipla, ASST papa Giovanni XXIII	Co-investigator	Acquisition of data
Lorenzo Capone	Centro Clinico delle Malattie Demielinizzanti dell’ASL di Biella - Ospedale degli Infermi di Biella	Co-investigator	Acquisition of data
Piero Nicolao	Reparto Di Neurologia, ULSS 1 Dolomiti, Ospedale Di Feltre	Co-investigator	Acquisition of data
Alessandra Lugaresi	UOSI Riabilitazione Sclerosi Multipla IRCCS - ISNB	Co-investigator	Acquisition of data
Augusto Rini	Centro Sclerosi Multipla - Ospedale A. Perrino	Co-investigator	Acquisition of data
Maria Merello	Azienda Socio Sanitaria Territoriale (A.S.S.T.) della Franciacorta	Co-investigator	Acquisition of data
Marta Bianchi	Ospedale Di Esine- Reparto di Neurologia	Co-investigator	Acquisition of data
Imma Plasmati	Centro SM c/o U.O. di Neurologia - P.O. Dimiccoli	Co-investigator	Acquisition of data
Renato Docimo	Centro Sclerosi Multipla - P.O. San Giuseppe Moscati	Co-investigator	Acquisition of data
Giovanna De Luca	Centro Sclerosi Multipla, Clinica Neurologica Policlinico SS. Annunziata	Co-investigator	Acquisition of data
Fiorella Mondino	Centro Sclerosi Multipla, SC Neurologia, ASO S.Croce e Carle	Co-investigator	Acquisition of data
Alessia Di Sapio	Centro SM - Ospedale Regina Montis Regalis	Co-investigator	Acquisition of data
Raffaella Clerici	Centro ad Alta Specializzazione per la diagnosi e la cura della sclerosi multipla - Ospedale Generale di zona Valduce	Co-investigator	Acquisition of data
Nerina Mascoli	Centro SM UO Neurologia ASST Lariana Ospedale S. Anna Como	Co-investigator	Acquisition of data
Maria Teresa Ferrò e Paola Grossi	Neuroimmunologia - Centro Provinciale per la diagnosi e terapia della Sclerosi Multipla, ASST Crema	Co-investigator	Acquisition of data
Davide Maimone	Centro Sclerosi Multipla - Osp. Garibaldi - Nesima	Co-investigator	Acquisition of data
Silvia Strumia	Ambulatorio Sclerosi Multipla della U.O. di Neurologia- sede di Forli’- AUSL della Romagna	Co-investigator	Acquisition of data
Maura Pugliatti	Centro Di Servizio E Ricerca Sulla Sclerosi Multipla AOU Di Ferrara	Co-investigator	Acquisition of data
Daniela Cargnelutti	SOC Neurologia - Day Hospital, ASUIUD P.O. S.Maria Della Misericordia - Udine	Co-investigator	Acquisition of data
Luisa Maria Caniatti	U.O.di Neurologia - Azienda ospedale universita’ S. Anna di Ferrara - Cona Ferrara	Co-investigator	Acquisition of data
Paola Crociani	Centro SM UO Neurologia, Fondazione IRCCS Casa Sollievo della Sofferenza, San Giovanni Rotondo (FG)	Co-investigator	Acquisition of data
Luca Massacesi	Centro di riferimento regionale per il trattamento della sclerosi multipla, SOD Neurologia II, AOU Careggi, Dipartimento Neuroscienze - Università di Firenze	Co-investigator	Acquisition of data
Susanna Malagù	Centro Sclerosi Multipla - U.O. di Neurologia - Ospedale Bufalini	Co-investigator	Acquisition of data
Giuseppe Ribizzi	U.O. Neurologia. Dipartimento Di Neuroscienze E Organi Di Senso - Ospedale Policlinico San Martino	Co-investigator	Acquisition of data
Simonetta Venturi	Ambulatorio Sclerosi Multipla - E.O. Ospedale Galliera	Co-investigator	Acquisition of data
Paola Gazzola	SC Neurologia - Ospedale P. Antero Micone - ASL 3 Genovese	Co-investigator	Acquisition of data
Nicola Renato Pizio	Ambulatorio Sclerosi Multipla - Neurologia ASL 4 Chiavarese	Co-investigator	Acquisition of data
Giampaolo Brichetto	Servizio di Riabilitazione AISM Liguria	Co-investigator	Acquisition of data
Katrin Plewnia	Centro SM USL Sudest, U.O.C. Neurologia, Osp. Misericordia	Co-investigator	Acquisition of data
Paolo Bellantonio	Centro Sclerosi Multipla - IRCCS Neuromed	Co-investigator	Acquisition of data
Roberto Balgera	Centro Sclerosi Multipla - Divisione di Neurologia - Azienda Ospedaliera A. Manzoni di Lecco	Co-investigator	Acquisition of data
Francesca De Robertis	Divisione di Neurologia - Ospedale ‘Vito Fazzi’	Co-investigator	Acquisition of data
Silvia Fermi	U.O Neurologia Ospedale Maggiore di Lodi	Co-investigator	Acquisition of data
Franco Fausto	Ambulatorio Sclerosi Multipla - Ospedale della Versilia	Co-investigator	Acquisition of data
Monica Mazzoni	Centro Malattie Disimmuni Del SNC E SNP Ospedale San Luca Lucca	Co-investigator	Acquisition of data
Giuseppe Meucci	Ambulatorio Sclerosi Multipla - Unita’ Operativa di Neurologia e Neurofisiopatologia, Spedali Riuniti di Livorno 10 padiglione II piano	Co-investigator	Acquisition of data
Elisabetta Cartechini	Centro Sclerosi Multipla - c/o UOC Neurologia - Ospedale di Macerata	Co-investigator	Acquisition of data
Guido Cavaletti	Centro di neuroimmunologia ASST MONZA E BRIANZA - Ospedale San Gerardo	Co-investigator	Acquisition of data
Maria Buccafusca	Centro Sclerosi Multipla - A.O.U. Policlinico Martino	Co-investigator	Acquisition of data
Placido Bramanti	IRCCS, Centro Neurolesi Bonino Pulejo	Co-investigator	Acquisition of data
Marzia Romeo	Centro Sclerosi Multpla - Ospedale San Raffaele	Co-investigator	Acquisition of data
Marco Rovaris	Centro SM, IRCCS Fondazione Don Carlo Gnocchi	Co-investigator	Acquisition of data
Marco Ronzoni	Centro SM - ASST-Rhodense, Garbagnate Milanese	Co-investigator	Acquisition of data
Torri Clerici Valentina	Fondazione IRCCS Istituto Neurologico Carlo Besta	Co-investigator	Acquisition of data
Luca Chiveri	Centro SM - Dipartimento Di Neuroscienze -ASST Ovest Milanese, Legnano (MI)	Co-investigator	Acquisition of data
Pierluigi Bertora	Centro Sclerosi Multipla - UO Neurologia - ASST FBF SACCO P.O. L. Sacco	Co-investigator	Acquisition of data
Simone Tonietti	Centro Sclerosi Multipla Ospedale San Carlo - ASST Santi Paolo e Carlo	Co-investigator	Acquisition of data
Milena De Riz	Centro Sclerosi Multipla- Fondazione IRCCS Ca′ Granda Ospedale Maggiore Policlinico di Milano	Co-investigator	Acquisition of data
Alessandra Protti	ASST GRANDE OSPEDALE METROPOLITANO NIGUARDA	Co-investigator	Acquisition of data
Patrizia Sola	Centro Malattie Demielinizzanti - Dipartimento Di Neuroscienze, Azienda Ospedaliero-Universitaria/OCSAE, UO Neurologia, Universita’ Di Modena E Reggio Emilia, Modena	Co-investigator	Acquisition of data
Mario Santangelo	U.O. C. Di Neurologia - AUSL - Modena, Ospedale Di Carpi	Co-investigator	Acquisition of data
Carlo Maremmani	Centro Sclerosi Multipla - Divisione Neurologica - Civico Ospedale di Carrara	Co-investigator	Acquisition of data
Gabriella Cacchio’	Ambulatorio dedicato Sclerosi Multipla, UO Neurologia AV5, Ospedale C. e G. Mazzoni	Co-investigator	Acquisition of data
Rosa Iodice	UOC Neurologia e Centro per l’Epilessia, Università Federico II di Napoli	Co-investigator	Acquisition of data
Michele Ragno	Ambulatorio Sclerosi Multipla - UO di Neurologia AV5 - Ospedale Civile Madonna del Soccorso- San Benedetto del Tronto	Co-investigator	Acquisition of data
Leonardo Sinisi	Centro SM, UOC Di Neurologia, Ospedale San Paolo, ASL Napoli 1 Centro, Napoli	Co-investigator	Acquisition of data
Vincenzo La Bua	CENTRO REGIONALE SCLEROSI MULTIPLA ….ADULTO - OSPEDALE PEDIATRICO ‘G. DI CRISTINA’ - ARNAS CIVICO - DI CRISTINA BENFRATELLI - PALERMO	Co-investigator	Acquisition of data
Roberto Cantello	Centro Sclerosi Multipla, Clinica Neurologica, Dipartimento di Medicina Traslazionale, Università del Piemonte Orientale	Co-investigator	Acquisition of data
Maria Luisa Piras	Centro diagnosi, cura e ricerca per Sclerosi Multipla - Ospedale S. Francesco - USL 3	Co-investigator	Acquisition of data
Salvatore Cottone	Centro Di Riferimento Regionale Per La Malattie Neurologiche a Patogenesi Immunitaria A.O.O.R. Villa Sofia-Cervello	Co-investigator	Acquisition of data
Luigi M. E. Grimaldi	Fondazione Istituto G. Giglio - Centro SM, Cefalu’	Co-investigator	Acquisition of data
Francesco Corea	Neurologia Centro SM - Ospedale San Giovanni Battista	Co-investigator	Acquisition of data
Giuseppe Santangelo	CENTRO REGIONALE SCLEROSI MULTIPLA IN ETA’ EVOLUTIVA - OSPEDALE PEDIATRICO ‘G. DI CRISTINA’ - ARNAS CIVICO - DI CRISTINA BENFRATELLI - PALERMO	Co-investigator	Acquisition of data
Paolo Immovili	Ospedale Guglielmo da Saliceto - UOC Neurologia	Co-investigator	Acquisition of data
Paolo Gallo	Centro Specializzato Regionale per la Sclerosi Multipla (CeSMuV) - Regione Veneto-Dipartimento Di Neuroscienze DNS-Azienda Ospedaliera - Universita’ degli Studi Di Padova	Co-investigator	Acquisition of data
Francesco D’Andrea	Centro SM - UO Neurologia - Casa Di Cura Villa Serena, Pescara	Co-investigator	Acquisition of data
Cristina Frittelli	Ambulatorioi Malattie Demielinizzanti - UOC Neurologia, Ospedale Lotti	Co-investigator	Acquisition of data
Livia Pasquali	Centro Malattie Demielinizzanti UO Neurologia, Dipartimento Di Medicina Clinica E Sperimentale, Universita’ Di Pisa	Co-investigator	Acquisition of data
Mario Falcini	Ospedale di Prato - Centro per la Sclerosi Multipla - Unita’ Operativa di Neurologia	Co-investigator	Acquisition of data
Franco Granella	Centro Sclerosi Multipla - Azienda Ospedaliero-Universitaria di Parma	Co-investigator	Acquisition of data
Ilaria Pesci	Centro SM UOC Neurologia Ospedale VAIO di Fidenza (PR), AUSL PR	Co-investigator	Acquisition of data
Anna Luisa Ancona	Ospedale San Jacopo di Pistoia Ambulatorio Malattie Demielinizzanti - Divisione Neurologica	Co-investigator	Acquisition of data
Umberto Aguglia	Ambulatorio Sclerosi Multipla - Grande Ospedale Metropolitano Bianchi Melacrino Morelli	Co-investigator	Acquisition of data
Roberto Bergamaschi	S.S. Sclerosi Multipla dell’IRCCS Fondazione Istituto Neurologico Nazionale C.Mondino	Co-investigator	Acquisition of data
Sara Montepietra	Centro SM, U.O.C. Neurologia, Ospedaliera Santa Maria Nuova, AUSL Reggio Emilia	Co-investigator	Acquisition of data
Antonello Giordano	S.C. Provinciale di Neurologia - ASP Ragusa - P.O. R. Guzzardi	Co-investigator	Acquisition of data
Mario Di Napoli	Centro Sclerosi Multipla - U.O. di Neurologia - Ospedale San Camillo De Lellis	Co-investigator	Acquisition of data
Silvia Romano	CENTERS Centro Neurologico Terapie Sperimentali - Sapienza Universita’ Di Roma, Azienda Ospedaliera S. Andrea	Co-investigator	Acquisition of data
Massimiliano Mirabella	UO Sclerosi Multipla, Fondazione Policlinico Universitario A. Gemelli IRCCS, Universita’ Cattolica Del Sacro Cuore	Co-investigator	Acquisition of data
Antonella Conte	Centro Clinico Policlinico Umberto I -Universita’ di Roma Sapienza	Co-investigator	Acquisition of data
Marco Peresson	Centro Clinico Sclerosi Multipla Ospedale Fatebenefratelli San Pietro	Co-investigator	Acquisition of data
Maria Grazia Grasso	Centro SM - Fondazione S. Lucia	Co-investigator	Acquisition of data
Elisabetta Ferraro	Centro SM - PO San Filippo Neri - ASL Roma 1, Roma	Co-investigator	Acquisition of data
Fioravante Capone	Centro SM - UOC Di Neurologia - Policlinico Universitario Campus Bio-Medico Di Roma	Co-investigator	Acquisition of data
Girolama Alessandra Marfia	UOSD Sclerosi Multipla, Policlinico Universitario Tor Vergata, Roma	Co-investigator	Acquisition of data
Daniela de Pascalis	Centro regionale per la diagnosi e cura della Sclerosi Multipla e malattie demielinizzanti, Osp. S. Eugenio di Roma	Co-investigator	Acquisition of data
Carlo Piantadosi	Centro Sclerosi Multipla - Az. Ospedaliera S. Giovanni - Addolorata	Co-investigator	Acquisition of data
Massimiliano Valeriani	Centro per la diagnosi e la cura delle malattie infiammatorie demielinizzanti in eta’ pediatrica - Ospedale Bambino Gesu’	Co-investigator	Acquisition of data
Vincenzo Busillo	U.O. di Neurologia- Ospedale Maria SS. Addolorata - Centro Diagnosi e Terapia Sclerosi Multipla	Co-investigator	Acquisition of data
Paolo Barone	Divisione Neurologica - Azienda Ospedaliera San Giovanni di Dio	Co-investigator	Acquisition of data
Monica Ulivelli	UOS Neuroimmunologia clinica- Ambulatorio Sclerosi multipla-AOUS	Co-investigator	Acquisition of data
Nicola De Stefano	Ambulatorio Sclerosi multipla - U.O.S.A. Malattie Neurodegenerative e Demielinizzanti - Azienda Ospedaliera Universitaria Senese	Co-investigator	Acquisition of data
Monica Ulivelli	UOC Neurologia e Neurofisiologia Clinica - Universita’ degli Studi di Siena	Co-investigator	Acquisition of data
Giuseppe Santuccio	Azienza Socio Sanitaria Territoriale (ASST) della Valtellina e Alto Lario - Sedi di Sondrio e Sondalo - Reparto di Neurologia	Co-investigator	Acquisition of data
Sergio Parodi	Centro Sclerosi Multipla e Malattie Demielinizzanti - Ospedale Civile S. Andrea - USL 5	Co-investigator	Acquisition of data
Sebastiano Bucello	Ospedale Muscatello di Augusta	Co-investigator	Acquisition of data
Sebastiano Traccis	Centro Prescrittore Sclerosi Multipla - U.O. di Neurologia - Presidio Ospedaliero di Ozieri - ASL N. 1 Sassari	Co-investigator	Acquisition of data
Roberto Zarbo	Centro Per La Diagnosi E Cura Della SM - Azienda Ospedaliero-Universitaria Di Sassari, Sassari	Co-investigator	Acquisition of data
Tiziana Tassinari	Centro SM Pietra Ligure S.C. Neurologia - Ospedale Santa Corona	Co-investigator	Acquisition of data
Fabio Bandini	Centro Sclerosi Multipla, Divisione di Neurologia, Ospedale S. Paolo	Co-investigator	Acquisition of data
Annamaria Marson	ASLTO4 - Neurologia - Ospedale Chivasso	Co-investigator	Acquisition of data
Paola Cavalla	Centro SM - Neurologia 1 D.U. - AOU Citta’ Della Salute E Della Scienza Di Torino	Co-investigator	Acquisition of data
Marinella Clerico	S.C.D.U. di Neurologia 1 - Azienda Ospedaliero Universitaria San Luigi Gonzaga	Co-investigator	Acquisition of data
Giulia De Rosa	Centro Sclerosi Multipla - Divisione Di Neurologia, Ospedale Civile, ASL 4	Co-investigator	Acquisition of data
Antonio Bertolotto	Centro Di Riferimento Regionale Per La SM (CRESM) - SCDO Neurologia - AOU San Luigi, Orbassano	Co-investigator	Acquisition of data
Daniele Imperiale	Centro Sclerosi Multipla - Divisione di Neurologia - Ospedale Maria Vittoria	Co-investigator	Acquisition of data
Paola Sarchielli	Centro Malattie Demielinizzanti - Ospedale *S. Maria* della Misericordia	Co-investigator	Acquisition of data
Maria Grazia Celani	Servizio Per Le Malattie Demielinizzanti - SC Di Neurofisiopatologia-Azienda Ospedaliera Di Perugia	Co-investigator	Acquisition of data
Bruno Marini	U.O.C. Neurologia,ULSS2- Marca Trevigiana-Regione Veneto - Ospedale S. Giacomo Apostolo, Castelfranco Veneto	Co-investigator	Acquisition of data
Marianna Fortunato	Ambulatorio Malattie Demielinizzanti - Unita’ Operativa Complessa di Neurologia - Ospedale Civile di Conegliano Veneto	Co-investigator	Acquisition of data
Mauro Zaffaroni	Centro Sclerosi Multipla, ASST Della Valle Olona, Ospedale Di Gallarate	Co-investigator	Acquisition of data
Davide Nasuelli	ASST Della Valle Olona Presidio Ospedaliero Di Saronno Ambulatorio Sclerosi Multipla U	Co-investigator	Acquisition of data
Paola Banfi	Centro Sclerosi Multipla, Ambulatorio Malattie Demielinizzanti - Ospedale di Circolo e Fondazione Macchi	Co-investigator	Acquisition of data
Andrea Mauro Brioschi	Istituto Auxologico Italiano IRCCS - Istituto Scientifico Ospedale S. Giuseppe e Ambulatorio	Co-investigator	Acquisition of data
Claudio Solaro	Dipartimento di Riabilitazione, CRRF “Mons. Luigi Novarese”	Co-investigator	Acquisition of data
Rocco Quatrale	Ambulatorio Sclerosi Multipla - Divisione di Neurologia - Ospedale dell’Angelo	Co-investigator	Acquisition of data
Patrizia Rossi	Ambulatorio Sclerosi Multipla e Malattie Demielinizzanti del SNC - UOC di Neurologia - Ospedale San Bassiano	Co-investigator	Acquisition of data
Alberto Gajofatto	Policlinico G.B. Rossi - Clinica Neurologica, Dipartimento di Neuroscienze, Biomedicina e Movimento	Co-investigator	Acquisition of data
Paolo Giannetti	Centro Sclerosi Mulpltipla - Ospedale Belcolle	Co-investigator	Acquisition of data
Vincenzo La Bua	Centro Sclerosi Multipla – Ospedale ARNAS Civico di Cristina Benfratelli	Co-investigator	Acquisition of data
Pierangelo Veggiotti	Centro Sclerosi Multipla Pediatrica – UOC Neurologia Pediatrica Ospedale V Buzzi, ASST Fatebenefratelli Sacco	Co-investigator	Acquisition of data
